# Nephrotoxic effect of heavy metals and the role of DNA repair gene among secondary aluminum smelter workers

**DOI:** 10.1007/s11356-022-24270-4

**Published:** 2022-11-23

**Authors:** Gehan Moubarz, Atef M. F. Mohammed, Inas A. Saleh, Eman M. Shahy, Mona A. Helmy

**Affiliations:** 1 Environmental & Occupational Medicine Department, Environment and Climate Change Research Institute, National Research Centre, Giza, Egypt; 2 Air Pollution Research Department, Environment and Climate Change Research Institute, National Research Centre, Giza, Egypt

**Keywords:** Heavy metal exposure, Secondary aluminum smelter, Kidney biomarkers, XPD gene

## Abstract

This study aims to estimate the association between some heavy metals in suspended particulate matter (SPM) and kidney damage among workers at different departments in a secondary aluminum production plant. It also investigates the association between Xeroderma Pigmentosum complementation group D (XPD) gene polymorphisms and worker’s susceptibility to kidney dysfunction. It was conducted on 30 workers from the administrative departments and 147 workers from different departments in the production line. Estimation of some heavy metals (Al, Co, Ni, Cu, Pb, and Cd) in suspended particulate matter (SPM) is done. Also, urinary levels of those metals were measured for all workers. Kidney injury molecule 1 (KIM-1), clusterin levels, and XPD protein level were estimated. Genotyping of XPD gene polymorphisms was performed. The measured annual average concentrations of the estimated heavy metals were lower than the permissible limits. Gravity area had the maximum concentration of metals with a higher Al average daily dose and hazardous index > 1. Kidney injury biomarkers (clusterin and KIM-1) were increased significantly (*p* < 0.05) while XPD protein showed the lowest levels among workers at the gravity and cold rolling areas. XPD *Asn/Asp* genotype was more dominant among those workers (85.7%). Conclusion: aluminum workers are at risk of kidney disorders due to heavy metal exposure. The individual’s susceptibility to the diseases is related to the DNA repair efficiency mechanisms. The defect in XPD protein represents a good indicator of susceptibility to the disease. KIM-1 and clusterin estimation is a predictor biomarker for early-staged kidney diseases.

**Supplementary Information:**

The online version contains supplementary material available at 10.1007/s11356-022-24270-4.

## Introduction

Heavy metals (HMs) have harmful effects on human health, and exposure to those metals has been increased by industrial activities and modern industrialization. Simultaneous exposure to metals may have cumulative effects (Ambade 2018; Gazwi et al. 2020; Morabito et al. 2020). It causes a serious environmental problem worldwide because of their toxicity, non-degradability, and widespread pollution as a result of industrial and concrete of growth (Liu et al. 2018; Ali et al. 2019; Malakootian et al. 2021). Although HMs are found throughout the earth’s crust, most environmental contamination and human exposure result from anthropogenic activities like mining and smelting operations (Javid et al. 2021). Moreover, HMs in scrap are released, as particulate (WHO 2019). The type and quality of scrap have a major effect on the significance of the emissions (Strater et al. 2010). Additionally, aluminum is thought to be the third most abundant element within the earth’s crust. In a secondary aluminum smelter, aluminum-bearing scrap was derived from mining operation (Katja 2010). The emissions of air pollutants may occur during the various stages of the aluminum production process (EEA 2016).

In general, heavy metal related to atmospheric particles may accumulate in an individual via main routes of exposure (inhalation and ingestion), which may cause adverse effects on human health (Amano and Ntiri-Asiedu 2020; Javid et al. 2021). Short and long-term exposures to those metals are capable of inducing non-carcinogenic and carcinogenic effects (ATSDR 2017). The harmful effects of HMs cause several diseases such as neurological, respiratory, liver, and kidney damage. Also, exposure to environmental pollutants may change protein synthesis, prevent DNA repair, and alter the stability of DNA (Moubarz et al. 2022). Particularly, the International Agency for Research on Cancer (IARC) has assigned several HMs, like Cd, Cr, and Ni, to the group of substances that are carcinogenic. Additionally, Pb and Co are suspected to be probable carcinogens (IARC 2014).

Also, HMs such as Al, Cd, and Pb are toxic metals which will cause kidney diseases in occupational settings. However, it has not been proved if these metals cause nephropathy even at low environmental exposure levels (Yan et al. 2020). Moreover, urinary kidney biomarkers such as kidney injury molecule 1 (Kim-1) and clusterin are early diagnostic biomarkers for renal injury by heavy metals (Zhou et al.^2016^). Evidence suggests that clusterin together with KIM-1 are two of the earliest markers of proximal tubular injury. However, clusterin has additional advantage of reflecting injury to multiple territories of the nephron including the distal tubule and collecting duct (Griffin et al. 2019). In addition, exposure to high levels of Al compounds leads to aluminum poisoning. Aluminum poisoning can prevent DNA repair. Recently, biomedical studies have established the fact that at very low quantity arsenic, nickel, and cadmium may stop the formation of individual DNA repair protein. It implies that human health is affected even at low doses (Hemmaphan and Bordeerat 2022). Gene polymorphisms are widely studied in expressions of genetic susceptibility to environmental pollutants. The study of genetic polymorphisms existing in DNA repair genes has also provided relevant susceptibility biomarkers to health outcomes in high-risk exposed individuals.

Most attention has focused on HMs, with their ability to cause renal damage. So, the goals of the current study were (i) to estimate some heavy metals (Al, Co, Ni, Cu, Pb, and Cd) in suspended particulate matter (SPM) collected from air of Al smelter, (ii) to evaluate non-carcinogenic risk assessment of investigated heavy metals by using the EPA model, and (iii) moreover to research the effect of exposure to the estimated elements on kidney damage with emphasis to the role of DNA repair gene (XPD) on worker’s susceptibility to such damage.

## Subjects and methods

### Study design

This study was conducted in two major aluminum factories in Helwan and El-Tebbin areas, Cairo, Egypt. In the two factories, aluminum is manufactured through secondary process by recycling aluminum scrap. During the process, the scrap is collected, isolated, and placed into a melting furnace to be molten at temperatures ranging from 1300 to 1400 degrees Fahrenheit. Aluminum raw materials are melted in casting ovens and poured into cold rolling mill, and then, the product is transferred to annealing oven area. After that, the product is transferred to gravity area to melt it and poured into different casting molds. The surface treatment of the sectors is finished by electrochemical method or by electrostatic painting in all colors. After that, final inspection is carried out and then packaged to be delivered internally or for export.

### Monitoring of heavy metals

- SPM samples were collected from the air at different working areas one day a week during the year 2020. The samples were collected using a calibrated vacuum pump at a rate of 10–12 L/minute for a period of 8 h (the shift duration of the workers). All samples were air-dried in dark place (Cheng et al. 2020; Hassan et al. 2020).
- The samples were transferred into vessel made from Teflon. 10 ml mixture of HNO_3_ and HCl (3:1) was added; after that, it was placed in an ultrasonic water bath (Ultra-sons H, J.P., Selecta, Spain) at 70 °C for 30 min. After digestion, we added 10 ml of water, and then, we filtered the resulting mixture. The solution was transferred to a volumetric flask and diluted to 25 ml. All the glassware and plastic vessels were rinsed by dilute (1:1) nitric acid for 24 h and then washed with water (Yuen et al. 2012; Mohammed et al. 2020; Pradhan and Ambade 2020). Metals (Cr, Ni, Cu, Pb, Al, and Cd) in SPM samples were determined by analytical method using inductively coupled plasma (ICP, Perkin Elmer).
- Health risk assessment model: the health risk model, developed by the Environmental Protection Agency of the United States (US EPA), was used in the current study to estimate the health risks of heavy metals in SPM. The workers were exposed to heavy metals via three primary pathways: inhalation of suspended particles (inhalation dose, *D*_*inh*_), ingestion of dust particles (ingestion dose, *D*_*ing*_), and skin dermal absorption of dust particles (skin dermal dose, *D*_*dermal*_). The average daily dose (*D*) was calculated for each exposure pathway (Moja and Mnisi 2013; Ambade and Sethi 2021). Exposure dose was expressed in terms of daily dose (mg/kg·day). The exposure factors used for calculation are shown in Table S1.
  1A $${\mathrm{D}}_{\mathrm{inh}}=\mathrm{C}\times \frac{{\mathrm{R}}_{\mathrm{inh}}\times \mathrm{EF}\times \mathrm{ED}}{\mathrm{PEF}\times \mathrm{BW}\times \mathrm{AT}}$$
  1B $${\mathrm{D}}_{\mathrm{ing}}=\mathrm{C}\times \frac{{\mathrm{R}}_{\mathrm{ing}}\times \mathrm{EF}\times \mathrm{ED}\times \mathrm{CF}}{\mathrm{BW}\times \mathrm{AT}}\times {10}^{-6}$$
  1C $${\mathrm{D}}_{\mathrm{dermal}}=\mathrm{C}\times \frac{\mathrm{SL}\times \mathrm{SA}\times \mathrm{ABS}\times \mathrm{EF}\times \mathrm{ED}\times \mathrm{CF}}{\mathrm{BW}\times \mathrm{AT}}\times {10}^{-6}$$
  1D $$\mathrm{The Average} \mathrm{Daily Dose}\left(\mathrm{D}\right)=\sum \left({\mathrm{D}}_{\mathrm{ing}}+{\mathrm{D}}_{\mathrm{inh}}+{\mathrm{D}}_{\mathrm{dermal}}\right)$$

According to standard EPA methods (Mohammed et al. 2022), the exposure risks to each metal were calculated into carcinogens and non-carcinogens as the following equations:

2A $$\mathrm{HQ}=\mathrm D/\mathrm{RfD}$$

2B $$\mathrm{HI}=\sum{\mathrm{HQ}}_{\mathrm{metals}}$$

2C $$\mathrm{CR}=\mathrm D\times\mathrm{CSF}$$

where.

*HQ*: hazard quotient (dimensionless, *HQ*<1).
*D*: average daily dose (mg/kg·day) for each metal.
*RfD*: reference dose (mg/kg·day) for exposure routes.
*HI*: hazard index (dimensionless). USEPA considers *HI*>1, mean adverse health effects occur due to metal exposure.
*CR*: cancer risk (dimensionless).
CSF: cancer slope factor (kg/mg·day). USEPA mentioned that cancer risk value should be (10^−6^<CR<10^−4^).

### Subjects

This study was conducted on 177 workers from the two selected factories: 30 workers from the administrative departments and 147 from production line in secondary aluminum smelter of the two included factories. The exposed workers were from the oven area, cold mill rolling area, gravity area, oxidation area, and painting area. The working duration of all the included workers was more than 5 years with working shift of 8 h/day.

### Questionnaire

After obtaining a written informed consent from all the included workers, they filled a detailed personal questionnaire including age, socioeconomic status, and smoking habit. Environmental and occupational present and past histories were registered (duration of exposure in years, shift duration in hours, and type of exposure). Workers with past history of kidney diseases and recurrent urinary tract infections were excluded.

### Biological monitoring

A first morning urine sample was collected in sterile cups from each worker. In addition, about 10 ml of blood samples was collected from all participants. The blood samples were divided into 2 aliquots. The primary aliquot was centrifuged for separation of serum to work out all serological tests. The second aliquot was collected in a vacationer containing EDTA as anticoagulant for DNA extraction and stored at − 80 °C until analysis.

The following analyses were preformed:

- Urinary concentrations of Al, Pb, Cd, Cu, Cr, and Ni were measured by inductively coupled plasma mass spectrometry (Agilent 7500 ce). In brief, each urine sample was diluted 20-fold with 1% nitric acid (60%; Wako, Osaka, Japan) and 2% 1-butanol (99.5%; Nacalai Tesque, Kyoto, Japan) and then filtered through a 0.45 μm pore membrane. Urinary creatinine concentrations were measured using a spectrophotometric method based on Jaffe’s reaction.
- Determination of urinary creatinine through the Jaffe reaction. The concentrations of urinary heavy metals (including urinary Pb, Cd, Cu, Ni, Cr, and Al) were calibrated on the basis of urinary creatinine and were reported as μg/g creatinine.
- Estimation of kidney function biomarkers: kidney injury molecule 1 (KIM-1) and clusterin (ng/ml) levels in serum by sandwich enzyme-linked immunosorbent assay (ELISA) according to manufacturer’s instruction (SinoGeneClon Biotech Co., Ltd., China).
- Estimation of Xeroderma Pigmentosum complementation group D (XPD) protein levels in serum by ELISA according to manufacturer’s instruction (SinoGeneClon Biotech Co., Ltd., China).
- Genotyping of XPD gene polymorphisms *Asp312Asn* in exon 10 (rs1799793) and *Lys751Gln* in exon 23 (rs13181) using polymerase chain reaction–restriction fragment-length polymorphism (PCR–RFLP) as described by Lainé et al. (2007).

Genomic DNA was isolated from blood samples collected from workers using the QIAamp DNA Blood Mini kit following the manufacturer’s protocol.

The polymorphisms were analyzed using the PCR–RFLP method. The PCR was performed in a total volume of 25 μl containing 100 ng of genomic DNA, 1× PCR Master Mix (Tiangen), and 5 pmol of forward primer (5-CTG TTG GTG GGT GCC CGT ATC TGT TGG TCT-3) and reverse primer (5-TAA TAT CGG GGC TCA CCC TGC AGC ACT TCC T-3) were added for XPD 10 amplification. In case of XPD 23 amplification, 5 pmol of forward primer (5-GCC CGC TCT GGA TTA TAC G-3) and reverse primer (5-CTA TCA TCT CCT GGC CCC C-3) were added. Amplifications were performed under the following conditions: 94°C for 4 min; 40 cycles of 94°C for 30 s (denaturation), 65°C for 30–45 s (annealing), and 72°C for 1–1.5 min (extension); followed by 72°C for 4 min. The obtained PCR products were digested with restriction enzyme, StyI and PstI, according to the manufacturer’s company protocol (Thermo Scientific, USA). The digested products were then separated on a 3% agarose gels (FMC Bioproducts) along with a 100–1500 bp DNA ladder. The appearance of 244 and 507 bp bands indicated the *Asp/Asp* genotype, while 474 plus 244 and 33 bp bands indicated the *Asn/Asn* genotype for XPD exon 10. The appearance of 290 and 146 bp bands indicated the *Lys/Lys* genotype, while 227 plus 146 and 63 bp bands indicated the *Gln/Gln* genotype for XPD exon 23.

### Statistical analysis

Data was tabulated and statistically evaluated on the Statistical Package of Social Science Software program, version 25 (IBM SPSS Statistics for Windows, Version 22.0, Armonk, NY: IBM Corp.). Data was obtainable using median and 25th and 75th percentiles for quantitative variables and frequency and percentage for qualitative ones. Comparison between groups was performed using the chi square or Fisher’s exact tests for qualitative variables. Quantitative was non-normally distributed variables, comparison between two groups was conducted using the Mann Whitney test, and comparison between more than two groups was conducted using the Kruskal Wallis test.

## Results

### Air monitoring results

**Fig. 1:**
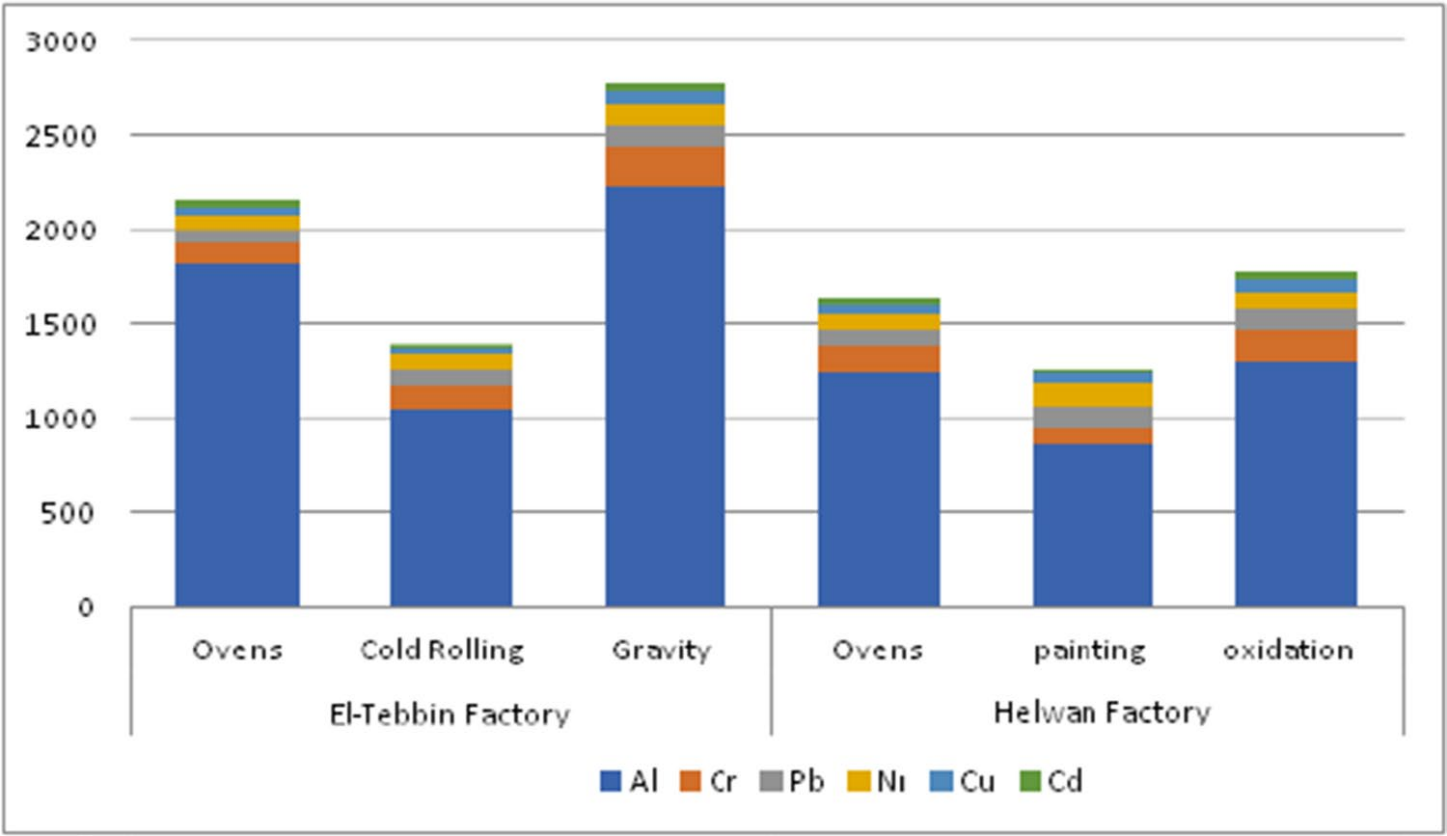
The annual average concentrations of individual heavy metals (μg/m^3^) at different investigated sites

The annual average concentrations of individual HMs (Cr, Ni, Cu, Pb, Al, and Cd) in SPM samples, collected during 2019 from El-Tebbin factory and Helwan factory, are represented in Fig. 1. Results showed that concentrations of individual HMs were higher in samples from Helwan factory than from El-Tebbin factory. The annual average levels of metals are in order Al > Cr > Pb > Cu > Ni > Cd.

Regarding sampling sites, the mean concentrations (μg/m^3^) of the measured metals at different sites were found to be in the range of Al (860–2236), Cr (84–205), Pb (67–120), Ni (73–124), Cu (30–60), and Cd (19–42). The concentrations of individual HMs were higher at gravity and oven areas than those in other partitions (Fig. 1).

**Fig. 2:**
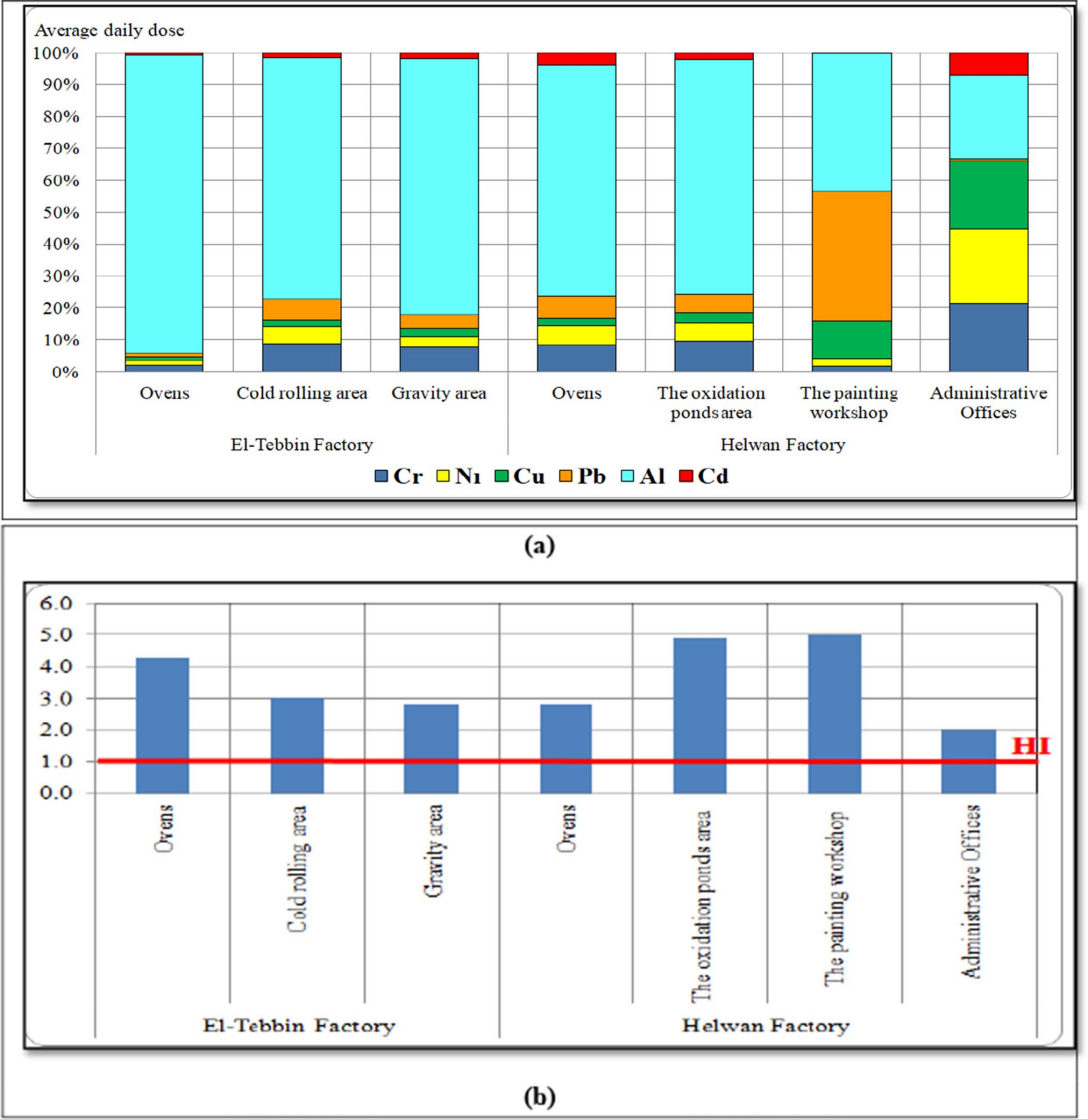
Contribution percentage in the average daily dose and the hazard index (*HI*) of individual heavy metal at each investigated site

Figure 2a shows that Al metal had a higher contribution percentages in the average daily dose at each investigated site. Figure 2b shows that hazard index (*HI*) values in investigated sites were higher than unity (*HI* > 1) suggested by US EAP (US EPA, 1989).

### Biological monitoring results

All workers included in this study were males, and their age was between 30 and 60 years with average 47.2 ± 9.5 years. They were employed for more than 5 years (21.3 ± 10.6 years). No variation in the smoking index was observed among all participants.

Table 1 shows that workers in oxidation, gravity, and oven areas had the highest creatinine-adjusted urinary concentrations of heavy metals especially for Al (13.4, 11.84, and 11 μg/g creatinine, respectively) and Pb (2.35, 2.2, and 1.78 μg/g creatinine, respectively). In addition, the highest urinary concentrations of Cd (0.18, 0.15 μg/g creatinine) were among the participants in oxidation and gravity areas, respectively. Regarding urinary Cu and Ni concentrations, they were the highest (3.6, 1.02 μg/g creatinine, respectively) among workers of the oxidation area. We observed also that the total urinary heavy metal concentration was the highest at the oxidation department followed by oven and gravity.

**Table 1:** Urinary metal concentration of Al, Cd, Cr, Cu, Ni, and Pb in all study participants and by industrial partition

Metals	Ovens	Cold rolling	Gravity	Painting	Oxidation	Administration	KS	*p* value
	Median (25th–75th)	Median (25th–75th)	Median (25th–75th)	Median (25th–75th)	Median (25th–75th)	Median (25th–75th)		
Al	11.84 (3.46–18)	7.03 (1.7–12.1)	11 (2.4–11.1)	4.7 (1.79–13.1)	13.4 (11.3–18.5)	10.3 (7.6–16.3)	9.306	0.09
Cd	0.14 (0.1–0.3)	0.14 (0.14–0.15)	0.15 (0.14–0.15)	0.14 (0.09–0.2)	0.18 (0.06–0.24)	0.08 (0.04–0.16)	15.756	0.01
Cr	0.85 (0.53–1.5)	0.6 (0.33–1.1)	0.54 (0.5–0.74)	0.6 (0.39–1.5)	1.2 (0.8–3.1)	0.85 (0.58–1.59)	8.112	0.15
Cu	2.5 (1.3–5)	3.2 (2.5–7)	4.2 (4–5)	3.3 (1.2–4.9)	3.6 (1.3–4.5)	1.8 (0.8–2.7)	12.893	0.02
Ni	0.85 (0.31–1.55)	0.49 (0.17–1.2)	0.48 (0–0.62)	0.5 (0.1–1.1)	1.02 (0.77–2)	0.78 (0.41–1)	11.581	0.04
Pb	2.2 (1.4–4.17)	1.75 (0.67–2.9)	1.78 (1.23–15.9)	1.47 (1.2–2.26)	2.35 (1.34–3.45)	1.65 (1.1–2.15)	7.305	0.19

Serum KIM-1 levels were increased significantly (*p* < 0.05) in workers at the gravity area. On the other hand, serum clusterin levels were significantly higher in workers from the cold rolling area (*p* < 0.05). In addition, the XPD protein showed the lowest levels among workers at gravity and cold rolling areas (Table 2).

**Table 2:** Comparison of some kidney biomarkers and XPD protein levels between workers at different industrial sites

	Ovens	Cold rolling	Gravity	Painting	Oxidation	Administration	KS	*p* value
	Median (25th–75th)	Median (25th–75th)	Median (25th–75th)	Median (25th–75th)	Median (25th–75th)	Median (25th–75th)		
KIM-1 (ng/ml)	9.7 (6–20)	12.85 (6–18.4)	31* (18–50.8)	12.75 (6.5–24.2)	9.25 (6–24.8)	10.2 (7.15–12.65)	13.88	0.016
Clusterin (ng/ml)	660 (86–1030)	749.5* (65–1000)	648 (520–1090)	728.5* (75–1102.5)	573 (80–777)	425.5 (375–532.5)	16.89	0.005
XPD protein	1825 (1150–2805)	1052 (262.5–2420)	885* (300–1115)	1143 (328.25–2480)	1460 (1155–2195)	2280 (1210–2960)	16.78	0.005

**Fig. 3:**
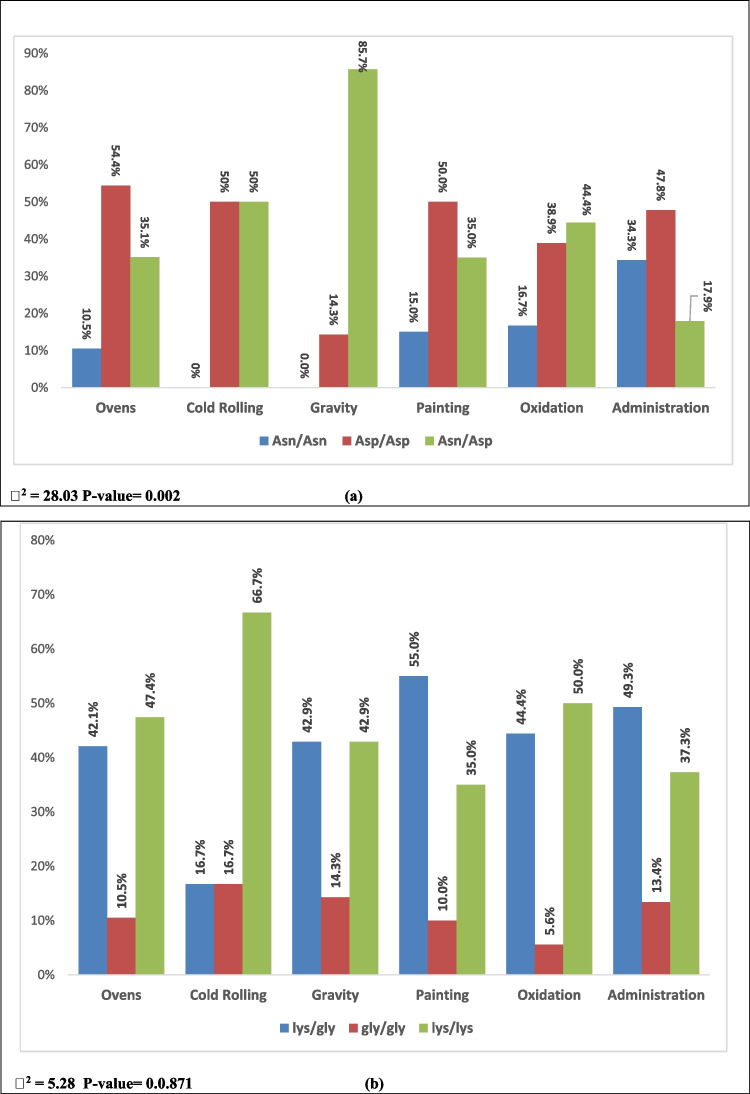
Frequency of XPD Asp312Asn and XPD Lys751Gln genotypes among the study workers

The current results showed significant difference in distribution of XPD Asp/Asn312 genotypes among workers (*p* < 0.05). The frequency of *Asn/Asp* genotype was significantly higher among workers in the gravity area compared to that in the other departments (Fig. 3a). Most of the workers on the gravity area (85.7%) have *Asn/Asp* genotype (Fig. 3a). In contrast, most of the workers in oven and administration areas had *Asp/Asp* genotype.

Among the workers, the frequency of the XPD Lys/Gln751 genotypes was variable; there is no significant difference between workers at different partitions in XPD Lys/Gln751 gene polymorphism distributions (*p* > 0.05; Fig. 3b).

## Discussion

Occupational exposure to heavy metals induces various adverse health risks (Garcia-Nino & Pedraza-Chaverri 2014). Emissions of air pollutants may occur during the different stages of the aluminum production process causing heavy metal-polluted workplace (EEA 2016). Exposure to high levels of HMs could lead to great risk of kidney damage. The present study is concerned with the occupational approach to investigate the kidney damage risk among workers in secondary aluminum production with emphasis to the role of XPD gene polymorphisms on susceptibility of workers to kidney diseases. In this study, the estimated annual Al and Pb metals have higher concentration in SPM than the other investigated metals; this may be attributed to industry processes. This is in accordance with Yuen et al. (2012). In contrast, Cd metal has the lowest concentration. Cd is a rare heavy metal. It occurs in combination with Zn (Moja and Mnisi 2013). The current results showed also that Cd, Pb, Cu, Ni, and Cr concentrations were below the permissible thresholds in Environmental Protection Law No. 4 of 1994 in the Arab Republic of Egypt for heavy metals from Al industry (EEAA, 1994). Workers were mainly exposed to those metals by inhalation fumes during the combustion process.

Gutowski et al. (2013) proved that huge supply of energy during aluminum industry affects pollutant concentrations. Particulate matter (PM) is a carrier of several elements such as Cd, Cu, Pb, and Co (Jena and Singh 2017). PM exposures in smelters are higher in concentration compared to fabrication (Neophytou et al. 2019). We observed that the total annual average concentrations of individual heavy metals were higher in gravity and oven areas than other partitions. The higher concentration levels may be attributed to higher temperature and combustion process which leads to increased emission of pollutants.

Additionally, our results showed that Al metal had a higher average daily dose at all production sites. Hazard index values were higher than unity (*HI* > 1) suggested by US EAP, which means that there is a higher risk probability for workers from exposure to heavy metals at all investigated sites. These results are in agreement with that reported by Mohammed et al. (2022).

Regarding urinary heavy metal concentrations, the analyzed heavy metals in this study are the most commonly toxic metals detected after occupational exposure. Our results showed that workers in oxidation, gravity, and oven areas of industrial process had the highest creatinine-adjusted urinary concentrations especially for Al, Cd, Cu, and Ni indicating that workers at these areas (the main steps in secondary aluminum smelter) may have a higher exposure risk to heavy metals.

The current results observed also that Al metal had higher contribution percentages in the average daily dose at all production sites. Continuously, the workers with higher urinary Al levels are at risk of diseases. Recently, Rahimzadeh et al. (2022) found that Al workers have high concentrations of urinary Al and at high risk of diseases.

The accumulation of Al in the kidney causes glomerular filtration failure and tubular kidney cell damage, leading to nephrotoxicity. In addition, Al can lead to acute renal glomerulonephritis, causing protein excretion in the urine (Al-Qhtani and Farran 2017). Our results observed an increase in KIM-1 levels among workers at cold rolling and gravity. Zhou et al. (2016) reported that KIM-1 may be an early diagnostic method of renal tubular injury in workers exposed to Pb metal. Low levels of some heavy metals can deposit in the kidney with long-term exposure and lead to kidney disease. Kim-1 is produced by injured cells of the proximal tubule. It may be a more specific marker of metal-induced proximal tubular injury (Prozialeck et al. 2007).

In addition, exposure to HMs may cause toxicity even at low concentrations. Such metal toxicity alternates gene expression of various genes that may cause an increase in susceptibility to several diseases. Although workers at cold rolling had a lower urinary Al levels, they were at risk of kidney damage. This may be attributed to low levels of XPD protein among these workers. On contrast, workers at oven and oxidation areas had a higher XPD protein and lower kidney damage even with the highest metal exposure. Recently, Singh et al. (2021) suggested that exposure to HMs is associated with DNA damage and DNA repair deficiency, leading to serious health problems in metal-exposed workers. Thus, Al workers who has a defect in DNA repair gene expression (XPD protein) are at more risk of developing kidney dysfunction.

Clusterin and Kim-1 were the most sensitive parameters for proximal tubular toxicity before any histopathological changes occurred (Vinken et al. 2012). Interestingly, our results found that workers at cold rolling had a high susceptibility to kidney damage due to their defect in DNA repair gene expression, whereas both kidney damage biomarkers KIM-1 and clusterin were higher in workers from cold rolling than oven areas. Clusterin upregulation has been observed with collecting duct and proximal tubule degeneration (Benjamin et al. 2019). Thus, workers with defect in DNA repair gene and exposed to some heavy metals in secondary Al smelter are at risk of kidney damage (proximal tubule and/or collecting duct).

Jadoon and Malik (2017) reviewed that many studies have shown that exposure to heavy metals is an essential source of DNA damage in human and animals. One of the most important defense mechanisms against the accumulation of DNA damage is the DNA repair genes, which includes XPD gene (Singh et al. 2021). The present results found that serum XPD levels (at acceptable exposure levels) could have a significant role on the development of kidney damage due to occupational exposure to heavy metals*.* Regarding the XPD gene polymorphisms, the current study detected significant differences in distribution of XPD Asp312Asn genotype between workers at different sites, as the *Asn/Asp* genotype was more dominant among the study workers at the gravity area (85.7%). While some studies have reported that the Asp/Asp of XPD 312 was associated with DNA damage (Seker et al. 2001), other studies showed that variant phenotypes have been associated with lower DNA repair capacity (Lunn et al. 2000). Similarly, in the present study, most of the gravity workers have *Asn/Asp* genotype, lower XPD protein, and a significant increase in kidney damage biomarker than workers at other sites. This means that workers who are carrying the *Asn/Asp* allele could be at high risk to develop kidney damage when exposed to heavy metals.

Additionally, there is no association between XPD Lys/Gln 751 gene polymorphism and the susceptibility to kidney damage. This finding is consistent with Corredor et al. (2020) that no previous reports linked kidney failure in humans with polymorphisms of this gene.

## Conclusion

Aluminum workers are at risk of kidney damage as a result of exposure to heavy metals even at levels below the standards. Workers were mostly exposed to those metals during smelting process. Aluminum workers are subjected to defect in DNA repair gene due to metal toxicity. This DNA repair defect will increase their susceptibility to kidney damage. We also provide evidence that XPD protein biomarker could be of great help in screening the susceptible workers who should be better removed from exposure. Also, KIM-1 and clusterin estimation can be used as a predictor biomarker for early-staged kidney diseases.

In summary, aluminum workers are at risk of developing kidney disorders even at levels below the standards. The susceptibility to the diseases is related to the DNA repair efficiency mechanisms of each individual.

## Supplementary Information

Comparison of heavy metal concentrations from different industrial areas around the world. Calculation of exposure daily dose due to heavy metals. Calculation of non-carcinogenic risk (hazard quotient and hazard index) due to exposure to heavy metals. Exposure factors used for calculation.

Supplementary file1 (DOCX 36 kb)

## Data Availability

The authors confirm that raw data are available from the corresponding author upon reasonable request.
