# Supplementary material for: Nephrotoxic effect of heavy metals and the role of DNA repair gene among secondary aluminum smelter workers
Source: Environ Sci Pollut Res Int. 2022 Nov 23;30(11):29814–23. doi: 10.1007/s11356-022-24270-4 (PMC9995418; doi:10.1007/s11356-022-24270-4)
Supplement: Supplementary file 1 — Supplementary file1 (DOCX 36 kb) [file 11356_2022_24270_MOESM1_ESM.docx]

**Table - 1S: Exposure factors used for calculation.**

| **Factor** | **Definition** | **Value** | **Unit** |
| --- | --- | --- | --- |
| **C** | Heavy metals concentrations | Measured | mg/m^3^ |
| **D _inh_** | The inhalation daily exposure dose | Calculated | mg/kg. day |
| **D _ing_** | The ingestion daily exposure dose |  |  |
| **D _dermal_** | The dermal daily exposure dose |  |  |
| **D** | The average daily dose |  |  |
| **BW** | Average body weight | 70 | kg |
| **R _ing_** | Ingestion rate | 100 | mg/day |
| **R _inh_** | Inhalation rate | 20 | m^3^/day |
| **PEF** | Particle emission factor | 1.36×10^9^ | m^3^/kg |
| **SA** | Surface areas of the skin that exposed to dust particles | 5700 | cm^-2^ |
| **SL** | Skin adherence factor | 0.07 | mg/m^3^ |
| **EF** | Exposure frequency | 180 | Days/year |
| **ED** | Exposure duration | 70 | Years |
| **AT** | Averaging time | 70 × 365 | Days |
| **ABS** | Dermal absorption factor | 0.001 | Unitless |
| **CF** | Conversion factor | 1×10^-6^ | Kg/mg |
| **RfD _inh_** | Inhalation reference dose | Cr (2.86E-05)  Ni (2.06E-02)  Cu (4.02E-02)  Cd (1.00E-03)  Pb (3.52E-03) | (mg/kg.day) |
| **RfD _ing_** | Ingestion reference dose | Cr (3.00E-03)  Ni (2.00E-02)  Cu (4.00E-02)  Cd (1.00E-03)  Pb (3.50E-03) |  |
| **RfD _dermal_** | Dermal reference dose | Cr (6.00E-05)  Ni (5.40E-03)  Cu (1.20E-02)  Cd (1.00E-05)  Pb (5.25E-04) |  |
| **CSF** | Cancer slope factor | Cr (42)  Ni (0.84)  Cd (6.3)  Pb (4.2) | (mg/kg.day^)-1^ |

Source: Mohammed et al., 2020; Mohammed et al., 2022.

**Table - 2S: Comparison of heavy metals concentrations form different industrial areas around the world (µg/m^3^).**

| Country | | Al | Cd | Cr | Cu | Pb | Ni | Ref. |
| --- | --- | --- | --- | --- | --- | --- | --- | --- |
| Egypt | El-Tabien Factory | 5116 | 102 | 425 | 135 | 265 | 272 | The current study |
|  | Helwan Factory | 4595 | 125 | 473 | 221 | 398 | 389 |  |
| Spain | industrial area | - | 0.32 | 10.9 | 262 | 13.1 | 2.29 | Jena and Singh, 2017 |
| Germany | Industrial area | - | 0.70 | 5.4 | 15.5 | 9.9 | 7.3 | Schaap et al., 2018 |
| Italy | Industrial area | 275 | 1 | 5 | 26 | 3 | 1 | Morabito et al., 2020 |
| India | Industrial area | - | 6.6 | 22.5 | 319 | 85.2 | 29.2 | Jena and Singh, 2017 |
| Taiwan | industrial area | - | 0.7 | 30.7 | 15.7 | 21.2 | 9.84 | Jena and Singh, 2017 |
| China | Industrial area-1 | - | 0.67 | - | - | - | 12.9 | Tian et al., 2015 |
|  | industrial area-2 | - | 0.52 | 9.6 | 130 | 46.7 | 9.56 | Jena and Singh, 2017 |
| Canada | Manitoba | 39 | - | - | - | - | - | ATSDR, 2008 |
| USA | Industrial area-1 | 8.0 | - | - | - | - | - | ATSDR, 2008 |
|  | industrial area-2 | - | 0.17 | 3.17 | - | 3.61 | 8.67 | Jena and Singh, 2017 |
|  | Chicago | 125 | - | - | - | - | - | ATSDR, 2008 |
| Brazil | Rio de Janeiro | 41 | - | - | - | - | - | ATSDR, 2008 |
| Egyptian Limits | | - | 2000 | 5000 | 20000 | 2000 | 5000 | EEAA, 1994 |

**Table - 3S: Exposure daily dose due to heavy metals**

| **Heavy metals** | | **Cr** | **Nı** | **Cu** | **Pb** | **Al** | **Cd** |
| --- | --- | --- | --- | --- | --- | --- | --- |
| **Dermal skin dose (D _dermal skin_), mg/kg.day** | | | | | | | |
| **El-Tabien Factory** | Ovens | 2.8E-07 | 2.1E-07 | 1.3E-07 | 1.9E-07 | 1.2E-05 | 1.2E-07 |
|  | Cold Rolling area | 3.4E-07 | 2.1E-07 | 8.4E-08 | 2.5E-07 | 3.0E-06 | 5.6E-08 |
|  | Gravity area | 2.8E-07 | 1.2E-07 | 9.3E-08 | 1.5E-07 | 2.9E-06 | 6.5E-08 |
| **Helwan Factory** | Ovens | 1.7E-07 | 1.2E-07 | 4.8E-08 | 1.4E-07 | 1.5E-06 | 7.9E-08 |
|  | The oxidation ponds area | 4.8E-07 | 2.8E-07 | 1.7E-07 | 2.8E-07 | 3.7E-06 | 1.1E-07 |
|  | The painting workshop | 2.4E-07 | 3.4E-07 | 1.7E-06 | 5.4E-06 | 6.1E-06 | 5.3E-08 |
|  | Administrative Offices | 1.5E-07 | 1.7E-07 | 1.6E-07 | 5.6E-09 | 1.9E-07 | 5.1E-08 |
| **Inhalation dose (D _Inhalation_), mg/kg.day** | | | | | | | |
| **El-Tabien Factory** | Ovens | 1.0E-08 | 7.6E-09 | 4.7E-09 | 6.9E-09 | 4.6E-07 | 4.4E-09 |
|  | Cold Rolling area | 1.2E-08 | 7.8E-09 | 3.1E-09 | 9.3E-09 | 1.1E-07 | 2.1E-09 |
|  | Gravity area | 1.0E-08 | 4.6E-09 | 3.4E-09 | 5.7E-09 | 1.1E-07 | 2.4E-09 |
| **Helwan Factory** | Ovens | 6.4E-09 | 4.4E-09 | 1.8E-09 | 5.2E-09 | 5.4E-08 | 2.9E-09 |
|  | The oxidation ponds area | 1.8E-08 | 1.0E-08 | 6.2E-09 | 1.0E-08 | 1.3E-07 | 4.0E-09 |
|  | The painting workshop | 8.7E-09 | 1.2E-08 | 6.1E-08 | 2.0E-07 | 2.2E-07 | 2.0E-09 |
|  | Administrative Offices | 5.7E-09 | 6.3E-09 | 5.8E-09 | 2.1E-10 | 7.0E-09 | 1.9E-09 |
| **Ingestion dose (D _Ingestion_), mg/kg.day** | | | | | | | |
| **El-Tabien Factory** | Ovens | 7.0E-05 | 5.1E-05 | 3.2E-05 | 4.7E-05 | 3.1E-03 | 3.0E-05 |
|  | Cold Rolling area | 8.5E-05 | 5.3E-05 | 2.1E-05 | 6.3E-05 | 7.4E-04 | 1.4E-05 |
|  | Gravity area | 6.9E-05 | 3.1E-05 | 2.3E-05 | 3.9E-05 | 7.3E-04 | 1.6E-05 |
| **Helwan Factory** | Ovens | 4.4E-05 | 3.0E-05 | 1.2E-05 | 3.5E-05 | 3.7E-04 | 2.0E-05 |
|  | The oxidation ponds area | 1.2E-04 | 7.0E-05 | 4.2E-05 | 7.0E-05 | 9.2E-04 | 2.7E-05 |
|  | The painting workshop | 5.9E-05 | 8.5E-05 | 4.1E-04 | 1.4E-03 | 1.5E-03 | 1.3E-05 |
|  | Administrative Offices | 3.9E-05 | 4.3E-05 | 3.9E-05 | 1.4E-06 | 4.8E-05 | 1.3E-05 |
| **Average daily dose (D), mg/kg.day** | | | | | | | |
| **El-Tabien Factory** | Ovens | 7.0E-05 | 5.1E-05 | 3.2E-05 | 4.7E-05 | 3.1E-03 | 3.0E-05 |
|  | Cold Rolling area | 8.5E-05 | 5.3E-05 | 2.1E-05 | 6.3E-05 | 7.4E-04 | 1.4E-05 |
|  | Gravity area | 6.9E-05 | 3.1E-05 | 2.3E-05 | 3.9E-05 | 7.3E-04 | 1.6E-05 |
| **Helwan Factory** | Ovens | 4.4E-05 | 3.0E-05 | 1.2E-05 | 3.5E-05 | 3.7E-04 | 2.0E-05 |
|  | The oxidation ponds area | 1.2E-04 | 7.0E-05 | 4.2E-05 | 7.0E-05 | 9.2E-04 | 2.7E-05 |
|  | The painting workshop | 5.9E-05 | 8.5E-05 | 4.1E-04 | 1.4E-03 | 1.5E-03 | 1.3E-05 |
|  | Administrative Offices | 3.9E-05 | 4.3E-05 | 3.9E-05 | 1.4E-06 | 4.8E-05 | 1.3E-05 |

**Table - 4S: Non-carcinogenic risk (Hazard quotient and hazard index) due to exposure to heavy metals**

| **Heavy metals** | | **Cr** | **Nı** | **Cu** | **Pb** | **Al** | **Cd** |
| --- | --- | --- | --- | --- | --- | --- | --- |
| **HQ  _dermal skin_** | | | | | | | |
| **El-Tabien Factory** | Ovens | 9.79E-03 | 1.02E-05 | 3.23E-06 | 5.40E-05 | - | 1.20E-04 |
|  | Cold Rolling area | 1.19E-02 | 1.02E-05 | 2.09E-06 | 7.10E-05 | - | 5.60E-05 |
|  | Gravity area | 9.79E-03 | 5.83E-06 | 2.31E-06 | 4.26E-05 | - | 6.50E-05 |
| **Helwan Factory** | Ovens | 5.94E-03 | 5.83E-06 | 1.19E-06 | 3.98E-05 | - | 7.90E-05 |
|  | The oxidation ponds area | 1.68E-02 | 1.36E-05 | 4.23E-06 | 7.95E-05 | - | 1.10E-04 |
|  | The painting workshop | 8.39E-03 | 1.65E-05 | 4.23E-05 | 1.53E-03 | - | 5.30E-05 |
|  | Administrative Offices | 5.24E-03 | 8.25E-06 | 3.98E-06 | 1.59E-06 | - | 5.10E-05 |
| **HQ _inhalation_** | | | | | | | |
| **El-Tabien Factory** | Ovens | 3.33E-06 | 3.80E-07 | 1.18E-07 | 1.97E-06 | - | 4.40E-06 |
|  | Cold Rolling area | 4.00E-06 | 3.90E-07 | 7.75E-08 | 2.66E-06 | - | 2.10E-06 |
|  | Gravity area | 3.33E-06 | 2.30E-07 | 8.50E-08 | 1.63E-06 | - | 2.40E-06 |
| **Helwan Factory** | Ovens | 2.13E-06 | 2.20E-07 | 4.50E-08 | 1.49E-06 | - | 2.90E-06 |
|  | The oxidation ponds area | 6.00E-06 | 5.00E-07 | 1.55E-07 | 2.86E-06 | - | 4.00E-06 |
|  | The painting workshop | 2.90E-06 | 6.00E-07 | 1.53E-06 | 5.71E-05 | - | 2.00E-06 |
|  | Administrative Offices | 1.90E-06 | 3.15E-07 | 1.45E-07 | 6.00E-08 | - | 1.90E-06 |
| **HQ _ingestion_** | | | | | | | |
| **El-Tabien Factory** | Ovens | 1.17E+00 | 9.44E-03 | 2.67E-03 | 0.0895 | - | 3.00 |
|  | Cold Rolling area | 1.42E+00 | 9.81E-03 | 1.75E-03 | 0.1200 | - | 1.40 |
|  | Gravity area | 1.15E+00 | 5.74E-03 | 1.92E-03 | 0.0743 | - | 1.60 |
| **Helwan Factory** | Ovens | 7.33E-01 | 5.56E-03 | 1.00E-03 | 0.0667 | - | 2.00 |
|  | The oxidation ponds area | 2.00E+00 | 1.30E-02 | 3.50E-03 | 0.1333 | - | 2.70 |
|  | The painting workshop | 9.83E-01 | 1.57E-02 | 3.42E-02 | 2.6667 | - | 1.30 |
|  | Administrative Offices | 6.50E-01 | 7.96E-03 | 3.25E-03 | 0.0027 | - | 1.30 |
